# Identification of the feature genes involved in cytokine release syndrome in COVID-19

**DOI:** 10.1371/journal.pone.0296030

**Published:** 2024-01-02

**Authors:** Bing Yang, Meijun Pan, Kai Feng, Xue Wu, Fang Yang, Peng Yang

**Affiliations:** The Second Affiliated Hospital, Guizhou University of Traditional Chinese Medicine, Guiyang, China; Albert Einstein College of Medicine, UNITED STATES

## Abstract

**Objective:**

Screening of feature genes involved in cytokine release syndrome (CRS) from the coronavirus disease 19 (COVID-19).

**Methods:**

The data sets related to COVID-19 were retrieved using Gene Expression Omnibus (GEO) database, the differentially expressed genes (DEGs) related to CRS were analyzed with R software and Venn diagram, and the biological processes and signaling pathways involved in DEGs were analyzed with GO and KEGG enrichment. Core genes were screened using Betweenness and MCC algorithms. GSE164805 and GSE171110 dataset were used to verify the expression level of core genes. Immunoinfiltration analysis was performed by ssGSEA algorithm in the GSVA package. The DrugBank database was used to analyze the feature genes for potential therapeutic drugs.

**Results:**

This study obtained 6950 DEGs, of which 971 corresponded with CRS disease genes (common genes). GO and KEGG enrichment showed that multiple biological processes and signaling pathways associated with common genes were closely related to the inflammatory response. Furthermore, the analysis revealed that transcription factors that regulate these common genes are also involved in inflammatory response. Betweenness and MCC algorithms were used for common gene screening, yielding seven key genes. GSE164805 and GSE171110 dataset validation revealed significant differences between the COVID-19 and normal controls in four core genes (feature genes), namely IL6R, TLR4, TLR2, and IFNG. The upregulated IL6R, TLR4, and TLR2 genes were mainly involved in the Toll-like receptor signaling pathway of the inflammatory pathway, while the downregulated IFNG genes primarily participated in the necroptosis and JAK-STAT signaling pathways. Moreover, immune infiltration analysis indicated that higher expression of these genes was associated with immune cell infiltration that mediates inflammatory response. In addition, potential therapeutic drugs for these four feature genes were identified via the DrugBank database.

**Conclusion:**

IL6R, TLR4, TLR2, and IFNG may be potential pathogenic genes and therapeutic targets for the CRS associated with COVID-19.

## Introduction

Coronavirus disease 19 (COVID-19) is an acute respiratory infectious disease caused by the novel coronavirus infection. The outbreak of the epidemic severely jeopardized the safety of the global population [1]. Fever, fatigue, and a dry cough represent the primary clinical manifestations. About half the patients developed dyspnea more than a week later, with rapid progression to acute respiratory distress syndrome, septic shock, refractory metabolic acidosis, and coagulation dysfunction in severe cases [2]. The damage to the body caused by viral infection is not limited to the virus itself [3]. A more serious effect is the systemic inflammatory reaction caused by the transitional immune system response to the virus, known as CRS. Several clinical studies have shown that CRS is an important cause of severe, critically illness, and even death due to COVID-19 [4, 5].

CRS refers to the excessive immune response caused by a dramatic increase in a large number of inflammatory cytokines [6]. Cytokines are soluble polypeptide proteins secreted by immune and non-immune cells, such as endothelial cells, epidermal cells, and fibroblasts, which play an intercellular regulatory role by binding corresponding receptors and controlling the inflammatory response, immune response, cell growth, differentiation, and maturation [7]. Cytokines can be divided into two categories according to the relationship between cytokines and inflammation. Class I represents pro-inflammatory cytokines, which can activate a variety of immune cells and promote inflammation. Class II includes an anti-inflammatory cytokine that neutralizes the effect of Class I cytokines, while the two interact to maintain immune system balance. In the case of severe lung infection in COVID-19 patients, cytokines can rapidly amplify the effect by binding to receptors, disrupting the homeostasis of the internal environment, leading to continuous lymphocyte and macrophage activation and amplification, and secreting a significant number of cytokines. This causes a series of pathological manifestations, such as endothelial dysfunction [8], systemic inflammatory response [9], coagulation dysfunction [10], and pulmonary fibrosis [11]. CRS is an important factor in the dangerous clinical manifestations of diseases, such as COVID-19, H5N1, and SARS, which can lead to death if not treated [12]. However, the CRS pathogenesis associated with COVID-19 remains unclear, while ideal intervention targets are lacking.

Disease occurrence and development is a complex biological process, which is often related to tens of thousands of genes and proteins. Therefore, it is extremely challenging to individually determine whether these genes are pathogenic using traditional methods. Bioinformatics is a new discipline involving the integration of life sciences, computer science, and informatics that uses massive biological data as the core and computers as tools for storage, retrieval, and analysis to reveal the related biological laws [13, 14]. In the big data era, a considerable amount of genetic disease data is stored in databases, the analysis of which helps to reveal the mechanisms underlying disease occurrence. This study conducts a secondary study on the COVID-19 data in the Gene Expression Omnibus (GEO) database by integrating bioinformatics. It also screens the CRS-associated disease genes from those in COVID-19 to provide an experimental basis for the pathogenesis and prevention of the CRS associated with COVID-19.

## Material and methods

### Data acquisition

The COVID-19-related dataset was retrieved from the Gene Expression Omnibus (GEO) database (https://www.ncbi.nlm.nih.gov/geo/). The training set information was obtained from the GSE164805 data of 10 COVID-19 patients and five healthy control patients (Healthy), while the validation set information was obtained from the GSE171110 data of 44 COVID-19 patients and 10 healthy control patients (Healthy). The dataset was downloaded using GEO query package (version 2.69.0) and the code used is listed in S1 and S2 in S1 Method. The CRS-related genes were searched in the GeneCards database (https://www.genecards.org/) using the keyword “Cytokine Release Syndrome”.

### Analysis of the differential expression genes (DEGs)

The Principal Component Analysis (PCA) was conducted using the factoMineR (version 2.9), Factoextra (version 1.0.7) and ggplot2 (version 3.4.3), and the code used is listed in S3 in S1 Method. The DEGs were screened using the Limma package (version 3.57.7) and #dplyr package (version 1.1.2), and the code is listed in S4 in S1 Method, with P<0.05 and |LogFC|>1 as conditions [15, 16]. DEG volcano map was created using the org.Hs.eg.db package (version 3.17.0), dplyr package (version 1.1.2) and ggplot2 package (version 3.4.3). The code is listed in S5 in S1 Method. A DEG heatmap was created using the pheatmap package (version 1.0.12), and the code used is listed in S6 in S1 Method.

### Common genes and their functional enrichment

The DEGs of the COVID-19 and CRS disease genes were used to construct a Venn diagram to obtain common genes, which were subjected to GO and KEGG enrichment analysis using the ggplot2 (version3.4.3), pathview (version 1.41.0), clusterProfiler (version 4.10.0) and org.Hs.eg.db (version 3.17.0) package. The code used is listed in S7 in S1 Method, while P<0.05 was used for screening [17, 18]. Visualization analysis was performed on the top-ranked results to better understand the association between COVID-19 and CRS.

### Analysis of the transcription factors regulating common genes

Transcription factors are essential for transcription regulation. Most transcription factors recognize and bind specific DNA sequences to regulate spatiotemporal target gene expression. Common gene transcription factor analysis is vital for exploring the regulation of complex biological processes. The key transcription factors responsible for regulating target genes were downloaded from the TRRUST (https://www.grnpedia.org/trrust) and hTFtaret (hust.edu.cn) databases [19]. The DEG and key transcription factor interaction network was constructed according to the transcription factor-target gene relationship using the Cytoscape 3.8.2 software and subsequently visualized.

### Feature gene acquisition

The common genes were imported into STRING 11.5 (https://cn.string-db.org/). The species was set to "Homo sapiens" (human) to construct and visualize a Protein-Protein Interaction (PPI) network. The results were exported in TSV format and imported into the Cytoscape 3.8.2 software to construct a PPI network diagram. The network was then topologically analyzed, and core genes were screened using the Betweenness and Matthews Correlation Coefficient (MCC) algorithms [20–22].

### Verification of the feature genes

The feature gene expression information of COVID-19 and the Healthy groups were retrieved from the GSE171110 datasets, while the differences between the two groups were compared via ggplot2 package (version 3.4.3). The code used is listed in S8 in S1 Method, employing Student’s t-test.

### The immune infiltration analysis of the feature genes

The ssGSEA algorithm in the GSVA package was used to quantify the immune cell proportion in the sample. The ssGSEA algorithm in the GSE164805 dataset was used for immune infiltration analysis, while the Wilcoxon test was employed to assess the immune infiltration expression differences between the COVID-19 and Healthy groups. The ccrrplot program and Spearman test were used to analyze the correlation between the feature genes and 28 infiltrating immune cells.

### Potential feature gene drug prediction

The DrugBank database (https://go.drugbank.com) is a comprehensive, free online resource. It contains detailed drug, drug-target, drug action, and drug interaction information of drugs and experimental drugs approved by the Food and Drug Administration (FDA), making it one of the most widely used reference drug resources in the world [23]. The DrugBank database was used to predict the core target regulatory drugs.

## Results

### Analysis of the COVID-19 and healthy DEGs

**Fig 1:**
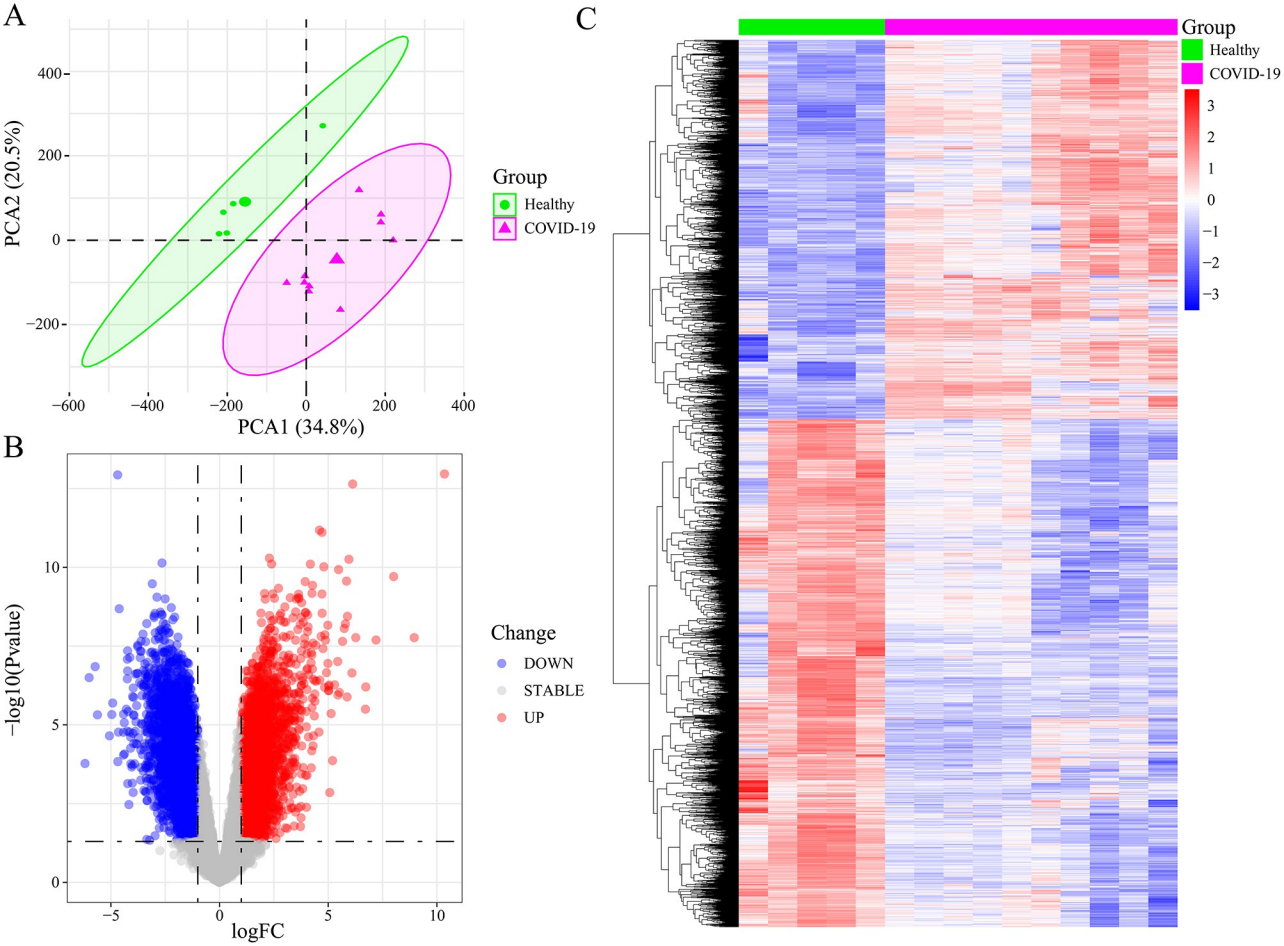
Analysis of the COVID-19 DEGs. (A) The Principal Component Analysis (PCA) of the COVID-19 (n = 10) and HC samples (n = 5). When PCA1 = 34.8% and PCA2 = 20.5%, COVID-19 and HC samples are clearly distinguished. (B) The 6950 DEGs volcano plots. The red dots indicate upregulated genes, the blue dots denote downregulated genes, and the gray dots represent non-DEGs, with FC≥1.0 and P-value<0.05. (C) The heatmaps showing the results of the clustering analysis based on 6950 DEGs.

Comprehensive gene expression information was obtained from 10 COVID-19 patients and five Healthy controls in the GSE164805 dataset. The PCA of these data sets showed a PCA1 of 34.8% and a PCA2 of 20.5%, suggesting differences between the two data groups, which prompted difference analysis (Fig 1A). A total of 6950 DEGs were screened according to parameters including P<0.05 and |LogFC|>1, of which 2969 were upregulated, and 3981 were downregulated (Fig 1B and 1C).

### Common gene acquisition and functional enrichment

**Fig 2:**
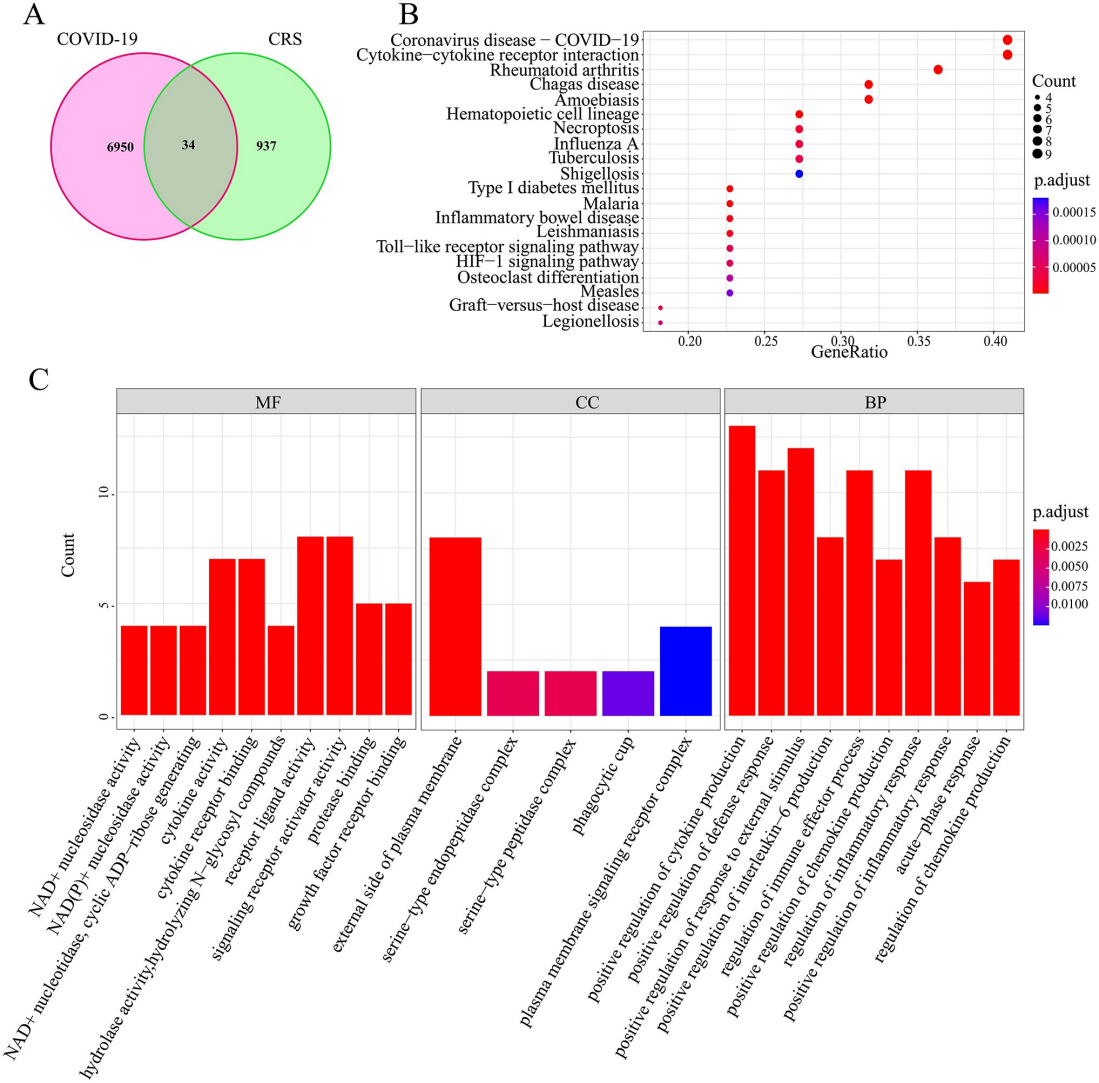
The common genes and their functional analysis. (A) The Venn diagram of the 6950 DEGs and 971 CRS genes. A total of 34 DEGs, namely common genes, were obtained. (B) The KEGG analysis of 34 common genes. (C) The GO analysis of 34 common genes.

A total of 971 CRS-related genes were obtained from the GeneCards database, which was crossed with DEGs to obtain 34 common genes (Fig 2A). The KEGG analysis results showed that these common genes were mainly enriched in Cytokine–cytokine receptor interaction, necroptosis, and Toll-like receptor signaling pathways (Fig 2B), indicating that the signaling pathways involved in common genes were primarily related to inflammatory factor production. The GO enrichment results show that the common genes mainly involve receiver ligand activity and signaling receiver activator activity in the molecular function (MF) module, external side of plasma membrane in the cellular component (CC) module, and positive regulation of cycline production, positive regulation of defense response and positive regulation of interleukin-6 production in the BP module (Fig 2C). However, the remaining 164805 DEGs enriched in the top five signaling pathways showed significant differences compared to the 34 common genes (S1 Fig and S1 Table). This suggested that these common genes were involved in multiple biological inflammatory response processes during COVID-19 progression.

### Analysis of the transcription factors for common gene regulation

**Fig 3:**
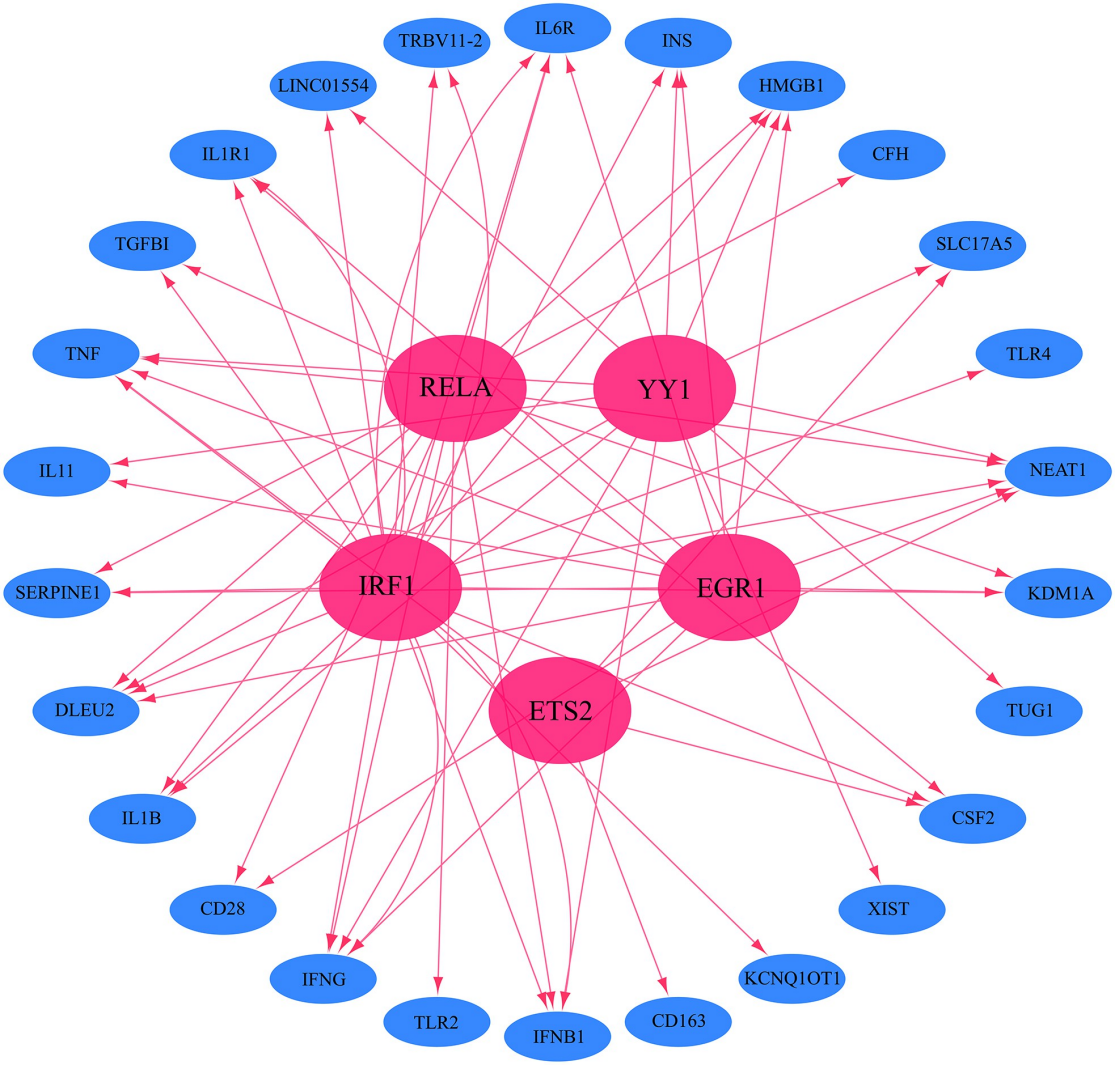
The network map of the common genes regulated by transcription factors. The 34 common genes are mainly regulated by 5 transcription factors RELA, YY1, IRF1, ETS2, and EGR1. Red represents the transcription factors, while blue denotes the common genes.

To further understand the role of common genes in the progression of COVID-19 to CRS, their regulatory transcription factors were analyzed. A total of 26 transcription factors were obtained from the TRRUST database, while 354 were acquired from the hTFtaret database. After intersection, five transcription factors were obtained, namely Yin Yang 1 (YY1), Homo sapiens v-rel avian reticuloendotheliosis viral oncogene homolog A (RELA), early growth response 1 (EGR1), v-ets erythroblastosis virus E26 oncogene homolog 2 (ETS2), and interferon regulatory factor 1 (IRF1). The network diagram of the transcription factors regulating common genes was visualized using the Cytoscape 3.8.2 software (Fig 3).

### Key gene screening

**Fig 4:**
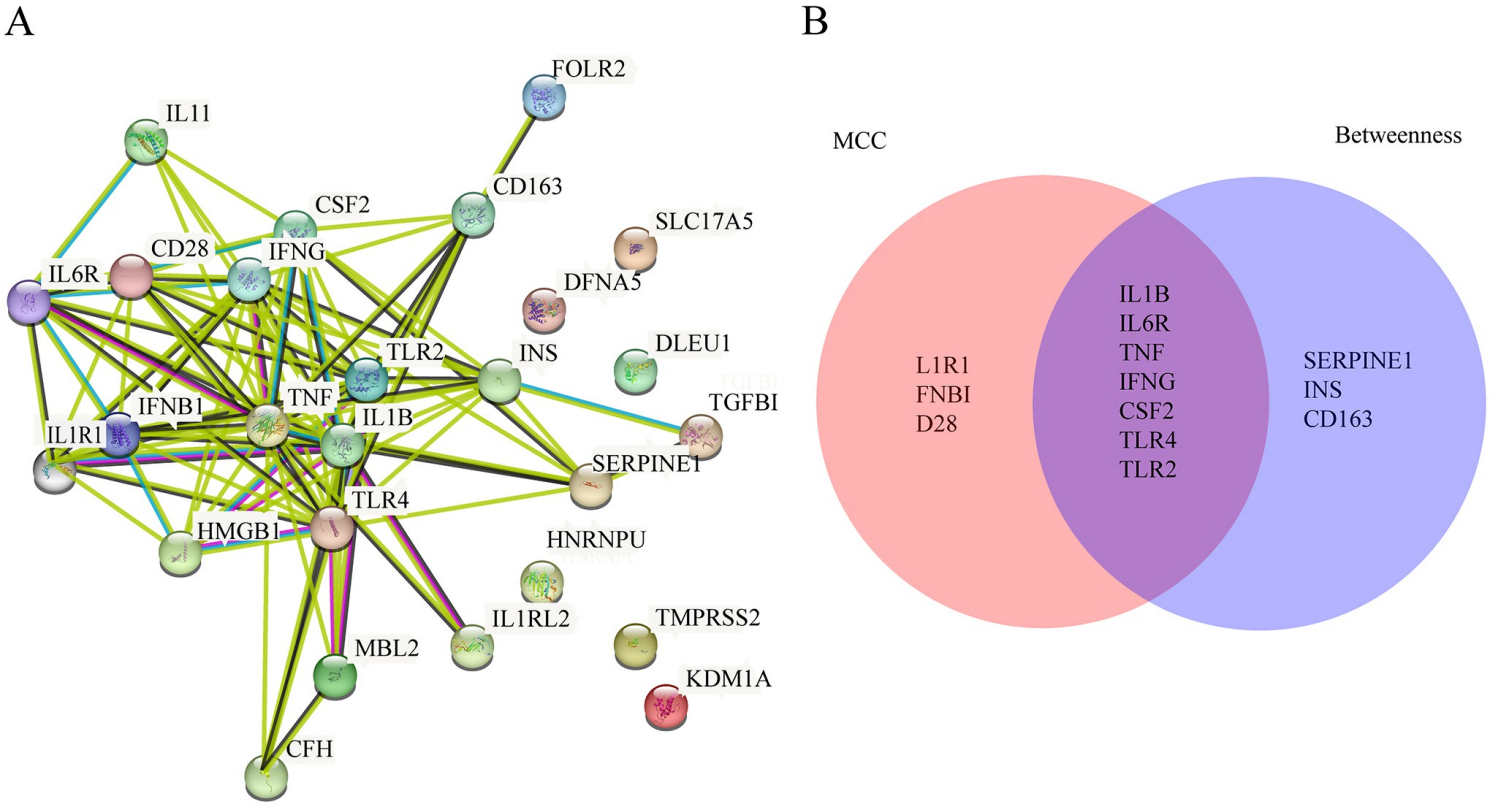
Key gene screening. (A) PPI network of common gene. There are 26 nodes with 92 edges, and the average degree value is 7.08. The size of the nodes in the figure and the depth of their colors are proportional to their degree value. (B)Venn diagram shows the intersection of the results of two algorithms.

In STRING11.5 analysis platform, the PPI network map was constructed using Cytoscape3.8.2 software, and the Betweenness and MCC algorithms were used to screen feature genes. (Fig 4A) shows the PPI network of common genes. The top 10 key genes obtained through the Betweenness algorithm are IL1 β, TNF, CD163, TLR4, INS, IFNG, CSF2, SERPINE1, TLR2, and IL6R. The top 10 key genes acquired by MCC algorithm are IL1 β, TNF, IFNG, CSF2, TLR4, TLR2, IFNB1, CD28, IL1R1, and IL6R. A total of 7 feature genes including IL1 β, IL6R, TNF, IFNG, CSF2, TLR4, and TLR2 were identified after crossing the two algorithms (Fig 4B).

### Verification of the key gene expression

**Fig 5:**
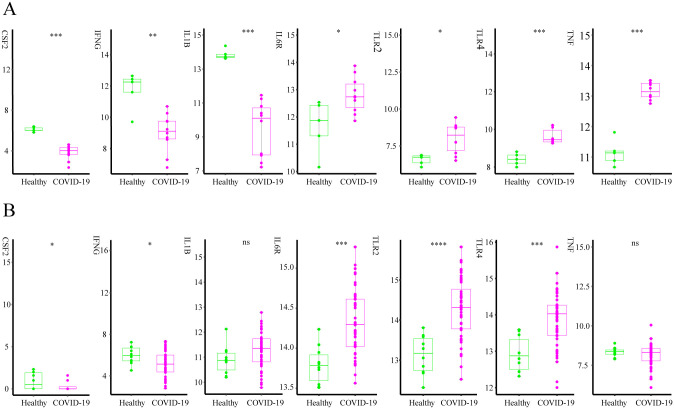
A comparison and validation between the key genes of the COVID-19 and healthy groups. (A) A histogram of the feature genes in the GSE164805 dataset. * indicates p<0.05, ** indicates p<0.01, and *** indicates p<0.001. (B) A histogram of the feature genes in the GSE171110 dataset. *indicates p<0.05, ***indicates p<0.001,and ****indicates p<0.0001.

The key feature genes were further screened by searching the database. In the GSE164805 dataset, seven genes significantly differed from the Healthy group. The CSF2, IFNG, and IL1B expression levels were considerably lower in the COVID-19 than in the Healthy group, while those of IL6R, TLR2, TLR4, and TNF were higher (Fig 5A). No significant differences were evident between the TNF and IL1B feature gene expression levels of the two groups in the GSE171110 dataset (Fig 5B), while CSF2 was not expressed in all COVID-19 samples. Therefore, the TNF, IL1B, and CSF2 genes were excluded. The remaining IL6R, IFNG, TLR4, and TLR2 genes (feature gene) were the most likely COVID-19 pathogenic genes that progressed into CRS.

### Analysis of the feature gene PPI network and KEGG enrichment

**Fig 6:**
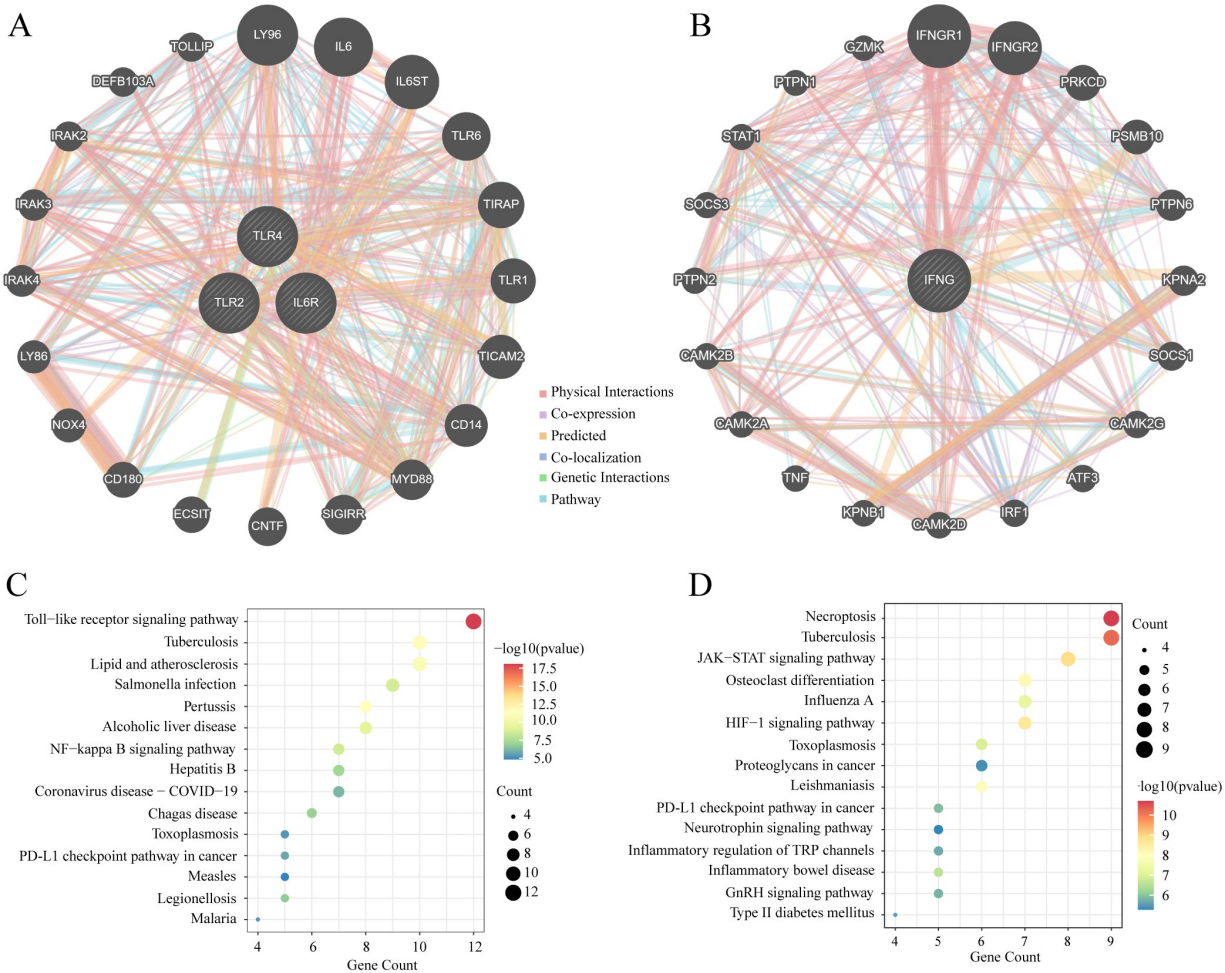
The PPI network and KEGG analysis of the feature genes. (A) The PPI network diagram of the upregulated genes. (B) The PPI network diagram of the downregulated genes. (C) The KEGG analysis of the upregulated and associated genes. (D) The KEGG analysis of the downregulated and associated genes.

The PPI network analysis was performed using the GeneMANIA database, while KEGG pathway analysis was conducted on the feature genes and their associated genes. (Fig 6A) shows the PPI network diagram of the upregulated IL6R, TLR4, and TLR2 genes, and (Fig 6B) shows that of the downregulated IFNG gene. The Toll-like receptor pathway was mainly involved in the upregulated and associated genes (Fig 6C), while the necroptosis and JAK-STAT signaling pathways were primarily involved in the downregulated IFNG and related genes (Fig 6D). These signaling pathways may be mainly related to the inflammatory response.

### Immune infiltration analysis

**Fig 7:**
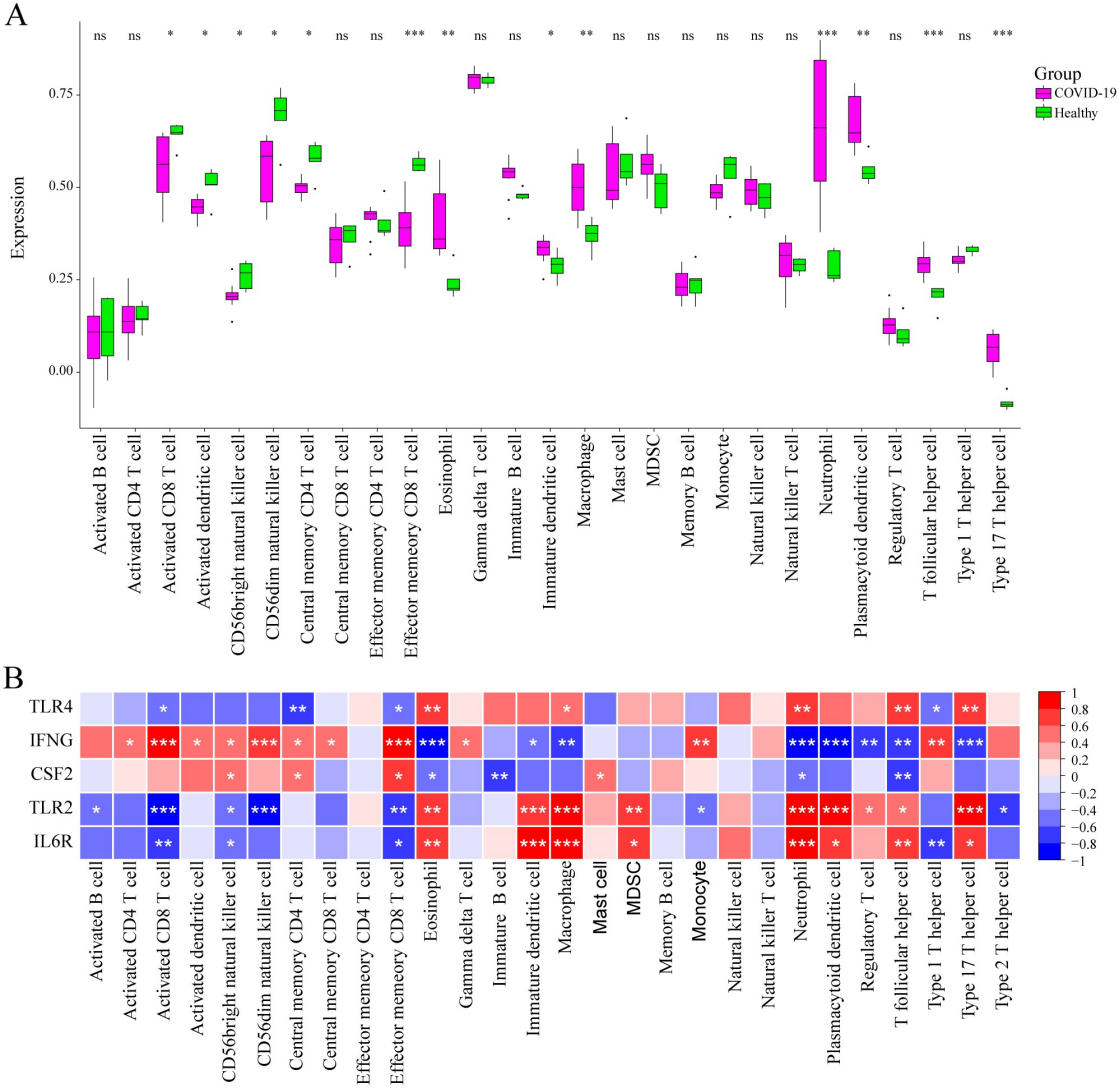
Immune infiltration analysis. (A) A comparison between the immune cell counts of the COVID-19 and healthy groups. * indicates p<0.05, ** indicates p<0.01, and *** indicates p<0.001. (B) The effect of higher feature gene expression on the immune cells. * indicates p<0.05, ** indicates p<0.01, and *** indicates p< 0.001.

The ssGSEA algorithm was used to analyze 27 types of immune cell infiltration to determine their impact on the feature genes. The results showed significant differences in the GSE164805 dataset between the COVID-19 and Healthy groups regarding 13 types of cells, including effector memory CD8^+^T cells, eosinophil, macrophages, neutrophils, plasmacytoid derivative cells, T follicular helper cells, and Type 17 T helper cells, P<0.05 (Fig 7A). The role of four feature genes in immune cell infiltration is shown in (Fig 7B). The higher TLR4 expression was positively correlated with eosinophil, macrophages, neutrophils, T follicular helper cells, and Type 17 T helper cells, and negatively with activated CD8^+^T cells, central memory CD4^+^T cells, and effector memory CD8^+^T cells. The higher IFNG expression was positively associated with activated CD8 T cells, activated dendritic cells, CD56bright natural killer cells, CD56dim natural killer cells, central memory CD4^+^T cells, and factor memory CD8^+^T cells, and negatively with eosinophil, macrophages, neutrophils, plasmacytoid dendritic cells, and Type 17 T helper cells. TLR2 was mainly positively correlated with eosinophil, immature dendritic cells, macrophages, neutrophils, plasmacytoid dendritic cells, and Type 17 T helper cells, and negatively with activated CD8^+^T cells, CD56dim natural killer cells, and factor memory CD8^+^T cells. The higher IL6R expression was positively associated with eosinophil, immature dendritic cells, macrophages, neutrophils, and T follicular helper cells and negatively with activated CD8^+^T cells. These results suggested that IL6R, TLR4, TLR2, and IFNG regulated multiple immune cell infiltration processes during COVID-19 progression.

### Prediction of potential therapeutic feature gene drugs

The DrugBank database was used to predict potential feature gene drugs for further analysis. The results showed that foreskin keratinocytes acted against IFNG, Tocilizumab inhibited IL6R, Eriodan restricted TLR4, and Adapalene impeded TLR4 Table 1.

**Table 1:** Analysis of potential drugs targeting key genes.

DRUGBANK ID	NAME	DRUG GROUP	ACTIONS	GENE
**DB06273**	Tocilizumab	approved	inhibitorantibody	IL6R
**DB11767**	Sarilumab	approved, investigational	antagonistantibody	IL6R
**DB15762**	Satralizumab	approved	binderantibody	IL6R
**DB01296**	Glucosamine	approved, investigational	inhibitor	IFNG
**DB05110**	VIR201	investigational	-	IFNG
**DB05111**	Fontolizumab	investigational	-	IFNG
**DB01250**	Olsalazine	approved	-	IFNG
**DB10770**	Foreskin fibroblast (neonatal)	approved	agonist	IFNG
**DB10772**	Foreskinkeratinocyte (neonatal)	approved	agonist	IFNG
**DB14724**	Emapalumab	approved, investigational	neutralizer	IFNG
**DB04933**	Eritoran	investigational	-	TLR4
**DB06447**	E5531	investigational	-	TLR4
**DB03017**	Lauric acid	approved, experimental	-	TLR4
**DB02767**	(R)-3-hydroxytetradecanoic acid	experimental	-	TLR4
**DB08231**	Myristic acid	experimental	-	TLR4
**DB01183**	Naloxone	approved, vet_approved	inhibitor	TLR4
**DB13615**	Mifamurtide	approved, experimental	ligand	TLR4
**DB11193**	Papain	approved	activator	TLR4
**DB00924**	Cyclobenzaprine	approved	inhibitor	TLR4
**DB00210**	Adapalene	approved	antagonist	TLR2
**DB05475**	Golotimod	investigational	-	TLR4/TLR2
**DB00045**	Lyme disease vaccine	approved, withdrawn	other/unknown	TLR2
**DB16474**	Pam2csk4	investigational	agonist	TLR2
**DB03963**	S-(Dimethylarsenic)Cysteine	experimental	-	TLR2
**DB11601**	Tuberculin purified protein derivative	approved	ligand	TLR2

## Discussion

This study analyzed a training set (GSE164805) to obtain a total of 34 CRS-related DEGs and four regulatory transcription factors from COVID-19 patients. After the validation set (GSE171110) analysis, only four genes, namely IL-6R, TLR4, TLR2, and IFNG, showed expression changes similar to those of the training set in COVID-19. Further analysis revealed that these four genes were key targets for inhibiting inflammatory factor production. These findings help reveal the CRS pathogenesis associated with COVID-19.

CRS is one of the main causes of deterioration and mortality in COVID-19 patients [24]. Although this study screened 34 genes associated with CRS (common genes) from COVID-19 patients, those primarily responsible for CRS progression and the role of different cytokines remain unclear. Enrichment analysis showed that these 34 genes were involved in multiple inflammatory pathways and differed significantly from the signaling pathways enriched by the remaining 164,805 DEGs, suggesting that they might play a key role in CRS progression. Furthermore, the results showed that the YY1, RELA, EGR1, ETS2, and IRF1 transcription factors regulated these common genes. However, the role of these transcription factors in the development of the CRS associated with COVID-19 and as targets in disease treatment remains unclear. Therefore, further research on the transcription regulatory function of these 34 common genes may be beneficial for elucidating the CRS mechanism associated with COVID-19.

The IL-6 ligand of IL-6R is vital for cytokine storm mediation. Binding to the IL-6R ligand can cause various biological effects by activating the Janus kinase (JAK) signal, including naïve T cell maturation into effector T cells, inducing the secretion of various cytokines and chemokines by vascular endothelial cells, and activating coagulation cascade reactions [25, 26]. Some studies have shown that IL-6 maintains elevated levels in the peripheral blood of COVID-19 patients over long periods [27, 28], possibly serving as a predictive biomarker for disease severity [29]. A retrospective observational study showed that treatment with the biological agent Tocilizumab, which targets IL-6R, was effective in 46.7% of critically ill COVID-19 patients [30]. However, the role of IL-6R in the CRS associated with COVID-19 and as a treatment target remains unclear. In this study, bioinformatics integration showed that the IL-6R gene expression level was significantly higher in COVID-19 peripheral blood monocytes than in the healthy controls. Predictive analysis indicated that the Tocilizumab, Sarilumab, and Satalizumab biological agents effectively alleviated the biological effect of IL-6R. These results elucidate the role of IL-6R in the CRS pathogenesis associated with COVID-19 and as a treatment target.

Toll-like receptors (TLRs) are key regulators of the innate immune system. It helps to identify self- and non-self-molecules, ultimately eliminating the non-self. Numerous studies have suggested that endosomal TLRs, mainly TLR3, TLR7, TLR8, and TLR4, play a role in CRS induction. TLR7/8 recognizes SARS-COV-2, and when it replicates to dsRNA, it is recognized by TLR3, driving inflammatory signaling, such as NF-κB and MAPK [31]. However, the role of TLR4 in the CRS associated with COVID-19 remains unclear. The persistently high interleukin-6 (IL-6) and tumor necrosis factor-α (TNF-α) levels in severe COVID-19 patients are important products of toll-like receptor 4 (TLR4) signaling. Therefore, believe that this viral infection and the subsequent organ damage may stimulate the toll-like receptor 4 (TLR4) pathways, increasing inflammatory cytokine production [32, 33]. Moreover, studies have shown that the TLR4 expression levels are significantly higher in the peripheral blood neutrophils and monocytes of severe COVID-19 patients than in healthy controls [31, 34]. This study indicated that the TLR4 expression levels were higher in the peripheral blood monocytes of COVID-19 patients with associated CRS than in healthy controls and revealed potential therapeutic drugs for targeting TLR4. These results confirm the role of TLR4 in the CRS pathogenesis associated with COVID-19 and its potential value as a treatment target.

Toll-like receptors 2 (TLR2), such as TLR4, belong to the TLRs protein family and can recognize β-defensins, heat shock and surfactant proteins, and high mobility group box 1 (HMGB1) proteins [35]. Studies have shown that the expression of TLR2 and its downstream MYD88 is correlated with COVID-19 severity and can sense the SARS-CoV-2 envelope protein to produce inflammatory cytokines [36]. Moreover, animal and in vitro cell experiments showed that the SARS-CoV-2 spike protein caused inflammation via TLR2-dependent NF-κB pathway activation [37, 38]. One of its major ligands, HMGB1, is significantly elevated in the serum of COVID-19 patients and is associated with disease severity and CRS development [39]. However, the TLR2 action mechanism in the CRS associated with COVID-19 requires further confirmation. The genomic analysis of different data sets showed significantly elevated TLR2 expression in the peripheral blood mononuclear cells of COVID-19 patients, while potential therapeutic drug analysis was performed for this target. These results help clarify the mechanism behind TLR2 occurrence in the CRS associated with COVID-19.

Interferon-gamma (IFNG) is produced by natural killer cells, macrophages, and innate immunity effector cells, as well as the Th1 and CD8^+^T cells that participate in the adaptative response [40]. IFNG displays antiviral, antitumor, and immunomodulatory properties. The role of IFNG in COVID-19 progression remains unclear. Although reports have shown that the serum IFNG level in patients with CRS associated with COVID-19 is significantly lower than that in healthy controls [41], the therapeutic effect of IFNG injection on patients with severe COVID-19 remains controversial [40]. This study indicated that IFNG gene expression in the peripheral blood mononuclear cells of patients with CRS associated with COVID-19 was significantly lower than in healthy controls. The drugs associated with this target were analyzed. These results help elucidate the role of IFNG in the development of the CRS associated with COVID-19.

In conclusion, this study revealed that IL-6R, TLR4, TLR2, and IFNG may be potential pathogenic genes in the CRS associated with COVID-19. These findings help clarify the CRS pathogenesis associated with COVID-19. However, given the data collection limitations in the database and the need for continuous improvement of the methods used for big data analysis, more large sample prospective studies are required to confirm these results.

## Conclusion

IL6R, TLR4, TLR2, and IFNG may be potential pathogenic genes and therapeutic targets for the CRS associated with COVID-19.

## Supporting information

S1 Method (DOC)

S1 Fig (TIF)

S1 Table Comparison of enrichment analysis between 34 DEGs and remaining 6916 DEGs. (DOC)

## References

[1] Khan M, Adil SF, Alkhathlan HZ, Tahir MN, Saif S, Khan M, et al. COVID-19: A Global Challenge with Old History, Epidemiology and Progress So Far. Molecules. 2020;26(1):39. doi: 10.3390/molecules26010039 33374759 PMC7795815

[2] Attaway AH, Scheraga RG, Bhimraj A, Biehl M, Hatipoğlu U. Severe covid-19 pneumonia: pathogenesis and clinical management. BMJ. 2021;372:n436. doi: 10.1136/bmj.n436 33692022

[3] Zhou F, Yu T, Du R, Fan G, Liu Y, Liu Z, et al. Clinical course and risk factors for mortality of adult inpatients with COVID-19 in Wuhan, China: a retrospective cohort study. Lancet. 2020;395(10229):1054–1062. doi: 10.1016/S0140-6736(20)30566-3 32171076 PMC7270627

[4] Gusev E, Sarapultsev A, Solomatina L, Chereshnev V. SARS-CoV-2-Specific Immune Response and the Pathogenesis of COVID-19. Int J Mol Sci. 2022;23(3):1716. doi: 10.3390/ijms23031716 35163638 PMC8835786

[5] Mohamed KL, Khosroshahi LM, Rokni M, Mokhtari T, Noorbakhsh F. Immunology, immunopathogenesis and immunotherapeutics of COVID-19; an overview. Int Immunopharmacol. 2021;93:107364. doi: 10.1016/j.intimp.2020.107364 33486333 PMC7784533

[6] Fajgenbaum DC, June CH. Cytokine Storm. N Engl J Med. 2020;383(23):2255–2273. doi: 10.1056/NEJMra2026131 33264547 PMC7727315

[7] Borish LC, Steinke JW. Cytokines and chemokines. J Allergy Clin Immunol. 2001;47(4):569–74. doi: 10.1067/mai.2003.108 12592293

[8] Bonaventura A, Vecchié A, Dagna L, Martinod K, Dixon DL, Tassell BWV, et al. Endothelial dysfunction and immunothrombosis as key pathogenic mechanisms in COVID-19. Nat Rev Immunol. 2021;21(5):319–329. doi: 10.1038/s41577-021-00536-9 33824483 PMC8023349

[9] Ye Q, Wang B, Mao J. The pathogenesis and treatment of the `Cytokine Storm’ in COVID-19. J Infect. 2020;80(6):607–613. doi: 10.1016/j.jinf.2020.03.037 32283152 PMC7194613

[10] Asakura H, Ogawa H. COVID-19-associated coagulopathy and disseminated intravascular coagulation. Int J Hematol. 2021;113(1):45–57. doi: 10.1007/s12185-020-03029-y 33161508 PMC7648664

[11] Hu J, Li Y, Chen X, Luo C, Zuo X. Pulmonary fibrosis and cytokine release syndrome after hyperactivation with sintilimab. J Clin Pharm Ther. 2020;45(6):1474–1477. doi: 10.1111/jcpt.13217 32662522

[12] Karki R, Kanneganti TD. The ’cytokine storm’: molecular mechanisms and therapeutic prospects. Trends Immunol. 2021;42(8):681–705. doi: 10.1016/j.it.2021.06.001 34217595 PMC9310545

[13] Ayyildiz D, Piazza S. Introduction to Bioinformatics. Methods Mol Biol. 2006;50(7):610–9. doi: 10.1007/978-1-4939-9442-7 31115882

[14] Zeti AMH, Shamsir MS, Tajul-Arifin K, Merican AF, Mohamed R, Nathan S, et al. Bioinformatics in Malaysia: hope, initiative, effort, reality, and challenges. PLoS Comput Biol. 2009;5(8):e1000457. doi: 10.1371/journal.pcbi.1000457 19714208 PMC2723929

[15] Liu H, Deng Z, Yu B, Liu H, Yang Z, Zeng A, et al. Identification of SLC3A2 as a Potential Therapeutic Target of Osteoarthritis Involved in Ferroptosis by Integrating Bioinformatics, Clinical Factors and Experiments. Cells. 2022;11(21):3430. doi: 10.3390/cells11213430 36359826 PMC9657506

[16] Chen JH, Wang LL, Tao L, Qi B, Wang Y, Guo YJ, et al. 2021. Identification of MYH6 as the potential gene for human ischaemic cardiomyopathy. J Cell Mol Med. 2021;25(22):10736–10746. doi: 10.1111/jcmm.17015 34697898 PMC8581323

[17] Wang Y. Identification of Ferroptosis-Related Genes in Alzheimer’s Disease Based on Bioinformatic Analysis. Front Neurosci. 2022;16:823741. doi: 10.3389/fnins.2022.823741 35197821 PMC8858973

[18] Wu Y, Jiang T, Hua J, Xiong Z, Chen H, Li L et al. Integrated Bioinformatics-Based Analysis of Hub Genes and the Mechanism of Immune Infiltration Associated With Acute Myocardial Infarction. Front Cardiovasc Med. 2022;9:831605. doi: 10.3389/fcvm.2022.831605 35463752 PMC9019083

[19] Zhang Q, Liu W, Zhang HM, Xie GY, Miao YR, Xia M, et al. hTFtarget: A Comprehensive Database for Regulations of Human Transcription Factors and Their Targets. Genomics Proteomics Bioinformatics. 2020;18(2):120–128. doi: 10.1016/j.gpb.2019.09.006 32858223 PMC7647694

[20] Parvin S, Sedighian H, Sohrabi E, Mahboobi M, Rezaei M, Ghasemi D, et al. Prediction of Genes Involved in Lung Cancer with a Systems Biology Approach Based on Comprehensive Gene Information. Biochem Genet. 2022;60(4):1253–1273. doi: 10.1007/s10528-021-10163-7 34855070

[21] Choi JY, Lim SY, Yun SY. [Knowledge Structure of Chronic Obstructive Pulmonary Disease Health Information on Health-Related Websites and Patients’ Needs in the Literature Using Text Network Analysis]. J Korean Acad Nurs. 2021;51(6):720–731. doi: 10.4040/jkan.21086 35023860

[22] Al-Antari MA, Al-Masni MA, Kim TS. Deep Learning Computer-Aided Diagnosis for Breast Lesion in Digital Mammogram. Adv Exp Med Biol. 2020;1213:59–72. doi: 10.1007/978-3-030-33128-3_4 32030663

[23] Wishart DS, Feunang YD, Guo AC, Lo EJ, Marcu A, Grant JR, et al. DrugBank 5.0: a major update to the DrugBank database for 2018. Nucleic Acids Res. 2018;46(D1):D1074–D1082. doi: 10.1093/nar/gkx1037 29126136 PMC5753335

[24] Que Y, Hu C, Wan K, Hu P, Wang R, Luo J, et al. Cytokine release syndrome in COVID-19: a major mechanism of morbidity and mortality. Int Rev Immunol. 2022;41(2):217–230. doi: 10.1080/08830185.2021.1884248 33616462 PMC7919105

[25] Kang S, Kishimoto T. Interplay between interleukin-6 signaling and the vascular endothelium in cytokine storms. Exp Mol Med. 2021;53(7):1116–1123. doi: 10.1038/s12276-021-00649-0 34253862 PMC8273570

[26] Kim JS, Lee JY, Yang JW, Lee KH, Effenberger M, Szpirt W, et al. Immunopathogenesis and treatment of cytokine storm in COVID-19. Theranostics. 2021;11(1):316–329. doi: 10.7150/thno.49713 33391477 PMC7681075

[27] Huang C, Wang Y, Li X, Ren L, Zhao J, Hu Y, et al. Clinical features of patients infected with 2019 novel coronavirus in Wuhan, China. Lancet. 2020;395(10223):497–506. doi: 10.1016/S0140-6736(20)30183-5 31986264 PMC7159299

[28] Chen N, Zhou M, Dong X, Qu J, Gong F, Han Y, et al. Epidemiological and clinical characteristics of 99 cases of 2019 novel coronavirus pneumonia in Wuhan, China: a descriptive study. Lancet. 2020;395(10223):507–513. doi: 10.1016/S0140-6736(20)30211-7 32007143 PMC7135076

[29] Gao Y, Li T, Han M, Li X, Wu D, Xu Y, et al. Diagnostic utility of clinical laboratory data determinations for patients with the severe COVID-19. J Med Virol. 2020;92(7):791–796. doi: 10.1002/jmv.25770 32181911 PMC7228247

[30] Luo P, Liu Y, Qiu L, Liu X, Liu D, Li J. Tocilizumab treatment in COVID-19: A single center experience. J Med Virol. 2020;92(7):814–818. doi: 10.1002/jmv.25801 32253759 PMC7262125

[31] Yapasert R, Khaw-On P, Banjerdpongchai R. Coronavirus Infection-Associated Cell Death Signaling and Potential Therapeutic Targets. Molecules. 2021;26(24):7459. doi: 10.3390/molecules26247459 34946543 PMC8706825

[32] Zhang YY, Ning BT. Signaling pathways and intervention therapies in sepsis. Signal Transduct Target Ther. 2021;6(1):407. doi: 10.1038/s41392-021-00816-9 34824200 PMC8613465

[33] Hariharan A, Hakeem AR, Radhakrishnan S, Reddy MS, Rela M. The Role and Therapeutic Potential of NF-kappa-B Pathway in Severe COVID-19 Patients. Inflammopharmacology. 2021;29(1):91–100. doi: 10.1007/s10787-020-00773-9 33159646 PMC7648206

[34] Aboudounya MM, Heads RJ. COVID-19 and Toll-Like Receptor 4 (TLR4): SARS-CoV-2 May Bind and Activate TLR4 to Increase ACE2 Expression, Facilitating Entry and Causing Hyperinflammation. Mediators Inflamm. 2021;2021:8874339. doi: 10.1155/2021/8874339 33505220 PMC7811571

[35] Root-Bernstein R. Innate Receptor Activation Patterns Involving TLR and NLR Synergisms in COVID-19, ALI/ARDS and Sepsis Cytokine Storms: A Review and Model Making Novel Predictions and Therapeutic Suggestions. Int J Mol Sci. 2021;22(4):2108. doi: 10.3390/ijms22042108 33672738 PMC7924650

[36] Zheng M, Karki R, Williams EP, Yang D, Fitzpatrick E, Vogel P, et al. TLR2 senses the SARS-CoV-2 envelope protein to produce inflammatory cytokines. Nat Immunol. 2021;22(7):829–838. doi: 10.1038/s41590-021-00937-x 33963333 PMC8882317

[37] Khan S, Shafiei MS, Longoria C, Schoggins JW, Savani RC, Zaki H. SARS-CoV-2 spike protein induces inflammation via TLR2-dependent activation of the NF-κB pathway. Elife. 2021;10:e68563. doi: 10.7554/eLife.68563.PMID 34866574 PMC8709575

[38] van der Sluis RM, Cham LB, Gris-Oliver A, Gammelgaard KR, Pedersen JG, Idorn M, et al. TLR2 and TLR7 mediate distinct immunopathological and antiviral plasmacytoid dendritic cell responses to SARS-CoV-2 infection. EMBO J. 2022;41(10):e109622. doi: 10.15252/embj.2021109622 35178710 PMC9108609

[39] Al-Kuraishy HM, Al-Gareeb AI, Alkazmi L, Habotta OA, Batiha GE. High-mobility group box 1 (HMGB1) in COVID-19: extrapolation of dangerous liaisons. Inflammopharmacology. 2022;30(3):811–820. doi: 10.1007/s10787-022-00988-y 35471628 PMC9040700

[40] Cremoni M, Allouche J, Graça D, Zorzi K, Fernandez C, Teisseyre M, et al. Low baseline IFN-γ response could predict hospitalization in COVID-19 patients. Front Immunol. 2022;13:953502. doi: 10.3389/fimmu.2022.953502 36225915 PMC9548596

[41] Mansoor S, Butt AR, Bibi A, Mushtaq S, Ullah I, Alshahrani F, et al. Expression of IFN-Gamma is significantly reduced during severity of covid-19 infection in hospitalized patients. PLoS One. 2023;18(9):e0291332. doi: 10.1371/journal.pone.0291332 37756264 PMC10530045

