# Supplementary material for: Identification of the feature genes involved in cytokine release syndrome in COVID-19
Source: PLoS One. 2024 Jan 2;19(1):e0296030. doi: 10.1371/journal.pone.0296030 (PMC10760774; doi:10.1371/journal.pone.0296030)
Supplement: S1 Method — (DOC) [file pone.0296030.s001.doc]

# **Supplementary methods** (code or scripts)

**1 ##### Data Expression Matrix in GSE164805 ####**

rm(list = ls())

setwd("")

library(GEOquery)##GEOquery version 2.69.0

gse_number = "GSE164805"

eSet <- getGEO(gse_number,

destdir = '.',

getGPL = F)

class(eSet)

length(eSet)

eSet = eSet[[1]]

exp <- exprs(eSet)

boxplot(exp)

clinica <- pData(eSet)

OK= identical(rownames(clinica),colnames(exp));

OK

if(!OK) exp = exp[,match(rownames(clinica),colnames(exp))]

gpl_number <- eSet@annotation

**2 #####GSE164805 grouping####**

Group=ifelse(str_detect(clinica$title,"healthy"),

"CON",

"COVID")

Group = factor(Group,

levels = c("CON","COVID"))

Group

a = getGEO(gpl_number,destdir = ".")

b = a@dataTable@table

probe = b[,c("ID","ORF")]

colnames(probe) = c("ID_probe","symbol")

**3 ####PC Aanalysis####**

pca=as.data.frame(t(exp))

library(FactoMineR) #FactoMineR version 2.9

library(factoextra) #factoextra version 1.0.7

library(ggplot2) #ggplot2 version 3.4.3

dat.pca <- PCA(pca, graph = FALSE)

pca_plot <- fviz_pca_ind(dat.pca,

geom.ind = "point",

col.ind = Group,

palette = c("#00F000", "#FC2947"),

addEllipses = T,

legend.title = "Groups"

pdf(file = "PCA.pdf",width=5,height = 4.5)

par(oma=c(3,3,3,3))

par(cex = 0.9,mar=c(6,5,4,3))

pca_plot

dev.off()

**4 #### DEGs analysis and volcano mapping ####**

library(limma) #limma version 3.57.7

library(dplyr) #dplyr version 1.1.2

design=model.matrix(~Group)

fit=lmFit(exp,design)

fit=eBayes(fit)

deg=topTable(fit,coef=2,number = Inf)

deg <- mutate(deg,ID_probe=rownames(deg))

probe = probe[!duplicated(probe$symbol),]

deg <- inner_join(deg,probe,by="ID_probe")

head(deg)

nrow(deg)

logFC_t=1

P.Value_t = 0.05

k1 = (deg$P.Value < P.Value_t)&(deg$logFC < -logFC_t)

k2 = (deg$P.Value < P.Value_t)&(deg$logFC > logFC_t)

change = ifelse(k1,

"DOWN",

ifelse(k2,

"UP",

"STABLE"))

table(change)

deg <- mutate(deg,change)

**5 library(clusterProfiler) #clusterProfiler version 4.10.0**

library(org.Hs.eg.db) #org.Hs.eg.db version 3.17.0

s2e <- bitr(deg$symbol,

fromType = "SYMBOL", toType = "ENTREZID",

OrgDb = org.Hs.eg.db)

dim(deg)

deg <- inner_join(deg,s2e,by=c("symbol"="SYMBOL"))

dim(deg)

length(unique(deg$symbol))

library(dplyr) #dplyr version 1.1.2

library(ggplot2) #ggplot2 version 3.4.3

dat = deg[!duplicated(deg$symbol),]

logFC_t=1

P.Value_t=0.05

p <- ggplot(data = dat,

aes(x = logFC,

y = -log10(P.Value))) +

geom_point(alpha=0.4, size=3.5,

aes(color=change)) +

ylab("-log10(Pvalue)")+

scale_color_manual(values=c("blue", "grey","red"))+

geom_vline(xintercept=c(-logFC_t,logFC_t),lty=4,col="black",lwd=0.8) +

geom_hline(yintercept = -log10(P.Value_t),lty=4,col="black",lwd=0.8) +

theme_bw()

pdf(file = "volcano plots.pdf",width=5,height =4.5)

par(cex = 0.5,mar=c(8,8,8,8))

p

dev.off()

**6 #####pheatmap####**

library(pheatmap) #pheatmap version 1.0.12

exp = exp[dat$ID_probe,]

rownames(exp) = dat$symbol

cg = dat[dat$change !="STABLE",]

length(cg)

n=exp[cg$symbol,]

dim(n)

annotation_col=data.frame(group=Group)

rownames(annotation_col)=colnames(n)

heatmap_plot <- pheatmap(n,

scale = "row",

annotation_col=annotation_col,

cluster_cols =F,

show_colnames = F,

show_rownames =F,

fontsize = 10,

fontsize_row=6,

color = colorRampPalette(c("blue","white","red"))(80)

)

pdf(file = "heatmap.pdf",width=5,height =8)

par(cex = 0.5,mar=c(8,8,8,8))

heatmap_plot

dev.off()

**7 #####wilcox.test####**

exp_unique <- as.matrix(exp)

group <- Group

pp_plus<-function(g){

library(ggplot2)

library(ggpubr)

library(ggsci)

frame<-data.frame(gene=g,group=group)

ggplot(data=frame,aes(x=group,y=gene,color=group))+

stat_boxplot(geom="errorbar",width=0.1,size=0.1,position=position_dodge(0.8),color="black")+#aes(color=group)

geom_boxplot(alpha=0.5,

size=0.1,

width=0.5,

#outlier.color = "blue",

#outlier.fill = "red",

outlier.shape=19,

outlier.size=0.1,

#outlier.stroke = NA,

position = position_dodge(0.8))+

scale_color_manual(values = c(CON = "#00F000", COVID= "#FF1493"),guide = guide_legend(reverse = TRUE))+

#scale_fill_manual(values = c(healthy = "yellow", COVID = "red"),guide = guide_legend(reverse = TRUE))+

stat_compare_means(aes(label=..p.signif..), label.x = 1.5, label.y = 15,method = "wilcox.test",size=5,color="black")+

geom_point(aes(color=group))+

theme_classic()#+

#theme(axis.title = element_text(size=18),axis.text = element_text(size=14)}

P<- pp_plus(exp_unique["TLR2",])

pdf(file = "TLR2.pdf",width=4,height =5)

par(cex = 0.5,mar=c(8,8,8,8))

P

dev.off()

**8 #####KEGG and GO enrichment ####**

library(ggplot2) #ggplot2 version 3.4.3

library(pathview) #pathview version 1.41.0

library(clusterProfiler) #clusterProfiler version 4.10.0

library(org.Hs.eg.db) #org.Hs.eg.db version 3.17.0

entrez_id_full_kegg = mapIds(x =org.Hs.eg.db,

keys =data$X1,

keytype = "SYMBOL",

column = "ENTREZID")

entrez_id_kegg = na.omit(entrez_id_full_kegg)

ENTREZID_kegg = data.frame(entrez_id_full_kegg)

KEGG_diff <- enrichKEGG(gene = ENTREZID_kegg$entrez_id_full_kegg,

organism = "hsa",

pvalueCutoff = 0.05,

qvalueCutoff = 0.05)

KEGG_diff <- setReadable(KEGG_diff, OrgDb = org.Hs.eg.db, keyType="ENTREZID")

pdf(file="KEGG.pdf",width = 10,height = 6)

dotplot(

KEGG_diff,

x = "GeneRatio",

color = "p.adjust",###

showCategory = 20,

label_format = 30)

dev.off()

GO=enrichGO(gene =ENTREZID_kegg$entrez_id_full_kegg,

OrgDb = org.Hs.eg.db,

pvalueCutoff =0.05,

qvalueCutoff = 0.05,

ont="all",

readable =T)

pdf(file="GO.pdf",width = 10,height = 15)

barplot(GO, drop = TRUE, showCategory =6,split="ONTOLOGY") + facet_grid(ONTOLOGY~., scale='free')

dev.off()
