# Supplementary figures and images for: Identification of the feature genes involved in cytokine release syndrome in COVID-19
Source: PLoS One. 2024 Jan 2;19(1):e0296030. doi: 10.1371/journal.pone.0296030 (PMC10760774; doi:10.1371/journal.pone.0296030)

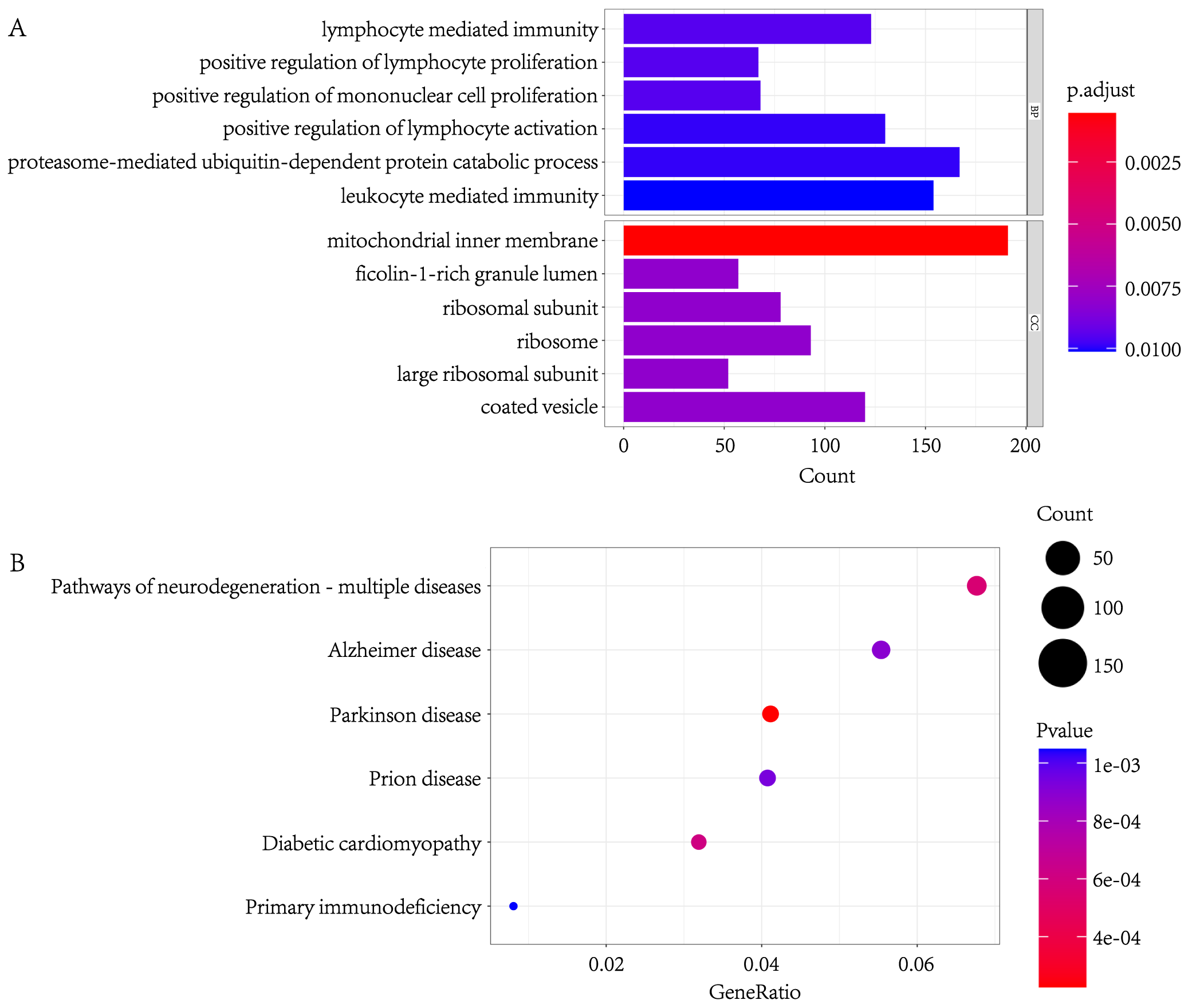

Supplement: S1 Fig — (TIF) [file pone.0296030.s002.tif]
