# Supplementary material for: Identification of the feature genes involved in cytokine release syndrome in COVID-19
Source: PLoS One. 2024 Jan 2;19(1):e0296030. doi: 10.1371/journal.pone.0296030 (PMC10760774; doi:10.1371/journal.pone.0296030)
Supplement: S1 Table — (DOC) [file pone.0296030.s003.doc]

**Supplementary Table l**

Table 1 Comparison of enrichment analysis between 34 DEGs and remaining 6916 DEGs

| **Group** | | **34 DEGs(Common gene)** | **geneID** | **remaining 6916 DEGs** | **geneID** |
| --- | --- | --- | --- | --- | --- |
| **GO**  **analysis** | **BP** | **positive regulation of cytokine production** | **TNF/CSF2/IL1B/TLR4/IL1RL2/INS/IFNG/CD28/HMGB1/TLR2/IL1R1/IL6R/SERPINE1** | **lymphocyte mediated immunity** | **FCER2/ULBP2/HLA-DOA/IL23A/PRDX1/CD55/HLA-DMB/IL20RB/MYO1G/CARD9/NCR3/CD1C/RAB27A/HLA-DMA/HLA-E/HLA-DPA1/MAP3K7/HLA-DRA/KMT5B/SHLD1/CD96/HPX/UNC93B1/CD40/CFI/IL13RA2/MALT1/FCGR3A/TREM2/MLH1/MYD88/C2/C7/CD40LG/TCIRG1/HLA-DPB1/KLRD1/TNFRSF1B/TAP2/B2M/C8A/SH2D1A/HAVCR2/CR1L/NCKAP1L/CLC/ZP3/NKG7/IRF7/RASGRP1/FOXP3/KDM5D/LYST/WAS/TFRC/ZBTB1/CEACAM1/RNF8/UNG/KIR3DL1/CD1D/BCL6/HLA-DQB2/FCER1G/MICA/CD27/IL18R1/TUBB/IL4/BTN3A2/PTPRC/HLA-A/NECTIN2/TRPM4/LGALS9/PAXIP1/AIRE/TRAF2/CLCF1/KLRC1/ATAD5/APLF/CADM1/KIR2DL4/NCR1/HLA-DRB1/SHLD3/NBN/HLA-DQB1/C1QB/EMP2/C17orf99/TLR8/CLEC12B/SLAMF1/TUBB4B/HMCES/CR1/ARL8B/C1R/FZD5/KLRC3/BTK/ULBP1/C1QBP/THOC1/HLA-DQA2/FADD/LAG3/CTSH/GATA3/PTPN6/CD74/BTN3A3/CD1A/SLAMF7/SMAD7/LILRB1/TREX1/SH2D1B/TBX21/KLRB1/AP1G1** |
|  |  | **positive regulation of interleukin-6 production** | **TNF/IL1B/TLR4/IL1RL2/IFNG/HMGB1/TLR2/IL6R** | **positive regulation of lymphocyte proliferation** | **RPS3/IL23A/CD55/HLA-DMB/CARD11/NCK1/IGF1/PYCARD/HLA-E/HLA-DPA1/IL13/TNFSF9/CD6/CD40/IL15/GPAM/MEF2C/RASAL3/FCGR3A/GPR183/MYD88/ADA/CD40LG/HLA-DPB1/SHH/CD276/HAVCR2/NCKAP1L/CD80/ZP3/RIPK2/TIRAP/EFNB1/NFATC2/FOXP3/EBI3/TFRC/IRS2/TLR9/CD1D/BCL6/PNP/IL4/CCL5/FGF10/PTPRC/HLA-A/CCDC88B/LGALS9/DHPS/CLCF1/VAV3/CD86/AIF1/ATAD5/ANXA1/SLAMF1/SELENOK/PELI1/BTK/TNFRSF13C/IGF2/FADD/IL1A/CD74/BCL2/HHLA2** |
|  |  | **regulation of immune effector process** | **TNF/CFH/IL1B/TLR4/INS/IFNG/CD28/HMGB1/MBL2/IFNB1/IL1R1** | **positive regulation of mononuclear cell proliferation** | **RPS3/IL23A/CD55/HLA-DMB/CARD11/NCK1/IGF1/PYCARD/HLA-E/HLA-DPA1/IL13/TNFSF9/CD6/CD40/IL15/GPAM/MEF2C/RASAL3/FCGR3A/GPR183/MYD88/ADA/CD40LG/HLA-DPB1/SHH/CD276/HAVCR2/NCKAP1L/CD80/ZP3/RIPK2/TIRAP/EFNB1/NFATC2/FOXP3/EBI3/TFRC/IRS2/TLR9/CD1D/BCL6/PNP/IL4/CCL5/FGF10/PTPRC/HLA-A/CCDC88B/LGALS9/DHPS/CLCF1/VAV3/CD86/AIF1/ATAD5/ANXA1/BCL2L1/SLAMF1/SELENOK/PELI1/BTK/TNFRSF13C/IGF2/FADD/IL1A/CD74/BCL2/HHLA2** |
|  |  | **positive regulation of chemokine production** | **TNF/IL1B/TLR4/IFNG/HMGB1/TLR2/IL6R** | **positive regulation of lymphocyte activation** | **ACTB/RPS3/HLA-DOA/IL23A/CD55/HLA-DMB/CARD11/NCK1/IGF1/PYCARD/HLA-DMA/HLA-E/HLA-DPA1/SOX12/IL13/HLA-DRA/ACTL6A/KMT5B/TNFSF9/SHLD1/CD6/CD40/IL15/MALT1/GPAM/MEF2C/RASAL3/FCGR3A/IHH/GPR183/MLH1/MYD88/MAP3K8/ADA/CD40LG/MDK/HLA-DPB1/SHH/CD276/ABL2/B2M/SH3KBP1/WNT10B/XRCC6/BAD/HAVCR2/NCKAP1L/BLOC1S3/CD80/DUSP10/ZP3/RIPK2/RASGRP1/TIRAP/EFNB1/NFATC2/FCHO1/FOXP3/DPP4/EBI3/ITPKB/TFRC/IRS2/ZBTB1/TLR9/PRKDC/CD1D/BCL6/HLA-DQB2/LYN/CD27/BRD7/FLOT2/PNP/IL4/CCL5/FGF10/PTPRC/HLA-A/CCDC88B/CCL21/LGALS9/PAXIP1/DHPS/RAG1/SIRPG/CLCF1/VAV3/CD86/AIF1/ATAD5/CCR7/HLA-DRB1/ANXA1/SHLD3/MMP14/VNN1/HLA-DQB1/SMARCD1/LGALS1/ICOS/SLAMF1/IL4R/SELENOK/HMCES/CD47/GAS6/PELI1/CR1/BTK/TNFRSF13C/PCID2/ZBTB16/IGF2/HLA-DQA2/FADD/RARA/IL1A/GATA3/PTPN6/AKIRIN2/CD74/SMARCA4/BCL2/HHLA2/LILRB1/RUNX3/TBX21/CSK/AP1G1** |
|  |  | **regulation of inflammatory response** | **TNF/IL1B/TLR4/IL1RL2/INS/IFNG/CD28/NEAT1/TLR2/IL1R1/SERPINE1** | **proteasome-mediated ubiquitin-dependent protein catabolic process** | **USP44/SGTA/RFFL/FBXL6/UBXN1/MDM2/ANAPC11/APC2/UMOD/TRIM25/UBXN11/PSMF1/TRAF4/RNF4/GID8/FBXL15/GSK3B/SIAH2/UBE2G2/TMUB1/ARIH2/TNFAIP1/HERPUD1/RMND5B/USP19/TOPORS/FBXL3/RNF185/NPLOC4/UBXN7/FBXL16/USP5/SIAH1/RNF34/RPL11/ANAPC16/FBXL12/ANAPC5/UBR1/DAB2/RNF103/SHH/PSMD4/SH3BGRL/USP9X/WNT10B/AGAP3/PSMC3/PSMA4/PSEN1/RNF175/SPSB3/RHBDD2/TAF1/TMEM129/RNF180/BIRC2/ZFAND2A/WWP2/HECTD3/SPSB4/PCBP2/ARAF/RNF122/KLHDC1/FHIT/CDC20/RAD23A/PSMA2/FBXO44/UBQLN4/PLK1/SPOPL/PRAME/ARIH1/PSMD11/FBXW5/FBXW4/NFE2L2/PSMB7/WWTR1/CSNK1D/RHOBTB3/ANAPC10/TRIM39/CUL1/RNF217/PJA2/RBCK1/BCAP31/PARK7/KBTBD6/HECTD1/KBTBD7/SPSB2/DERL3/FBXL7/ZSWIM8/TMUB2/NEDD4L/FBXO2/TRIP4/PANO1/ERLEC1/UFL1/DDA1/PSMB1/UFD1/ASCC2/UBE2W/DET1/RNF19A/AMFR/UCHL1/FBXO3/EDEM3/ZNRF1/PSMC1/RNF5/BTRC/ANKIB1/UBQLN3/KLHL42/TRIM3/ARRB1/RNF144A/SDCBP/PSMC2/PSMD7/KLHL20/SH3RF3/CDC34/RAD23B/TBL1XR1/PELI1/TRIM71/PSMA7/HSPA1B/GIPC1/FBXL19/UBXN2B/TRIB1/ASCC3/NSFL1C/MAN1A1/DDRGK1/IL33/UBE3D/PSMD10/AKIRIN2/RBX1/OS9/KLHL40/DMAC2/PIAS1/PPP2CB/SMAD7/STUB1/FOXRED2/FBXW11/TBX21/FBXL8/DNAJB9/YOD1/PSMA1/ASB2/FZR1** |
|  | **CC** | **external side of plasma membrane** | **TNF/TLR4/CD28/CD163/FOLR2/MBL2/IL1R1/IL6R** | **ficolin-1-rich granule lumen** | **CTSZ/CFD/COMMD3/TNFAIP6/VCL/KCMF1/AGL/SRP14/CST3/ATG7/LTA4H/ACTR2/C1orf35/HBB/CSNK2B/DYNLT1/COTL1/OSTF1/EEF2/PGM1/CAPN1/ALDOA/CAT/XRCC6/PSMC3/GNS/HSP90AA1/MMP9/DERA/CSTB/PSMA2/IMPDH2/PSMD11/ARSB/PSMB7/GSTP1/PNP/PSMB1/LRG1/FGL2/KRT1/JUP/PSMC2/PSMD7/ACTR10/PFKL/HSPA1B/FCN1/YPEL5/PPIA/GUSB/NME2/QPCT/CTSH/CRISPLD2/MNDA/TIMP2** |
|  |  | **serine-type endopeptidase complex** | **CFH/MBL2** | **ribosomal subunit** | **RPS3/RPL35A/RPL18A/RPS2/RPL13/GADD45GIP1/MRPS34/MRPL11/RPL37/RPL28/RPSA/RPL7A/RPL3L/AURKAIP1/ZCCHC17/RPL41/MRPL38/MRPL23/RPL19/MRPL55/RPS17/MRPS2/MRPL21/MPV17L2/MRPS15/MRPL14/EIF2A/RPS4X/RPS16/RPL11/RPS28/MRPL34/UBA52/FXR2/MRPL54/MRPL24/MRPS18C/RPL24/MRPL41/MRPS6/RPL35/MRPL45/RPL17/RPS23/NSUN3/MTERF4/MRPS35/MRPL18/RPL23/RPS18/RPL34/DAP3/RPL22/RPL26/MRPS21/RPL5/RPS20/MRPS12/RPL36/RPS21/RPL10L/RPL39/MRPL46/RPL13A/MRPL30/MRPS9/RPL4/RPS27/RPS11/MRPL1/RPL30/RPL10A/RPS4Y1/MRPL53/MRPL4/MRPL13/MRPL39/RPL23A** |
|  |  | **serine-type peptidase complex** | **CFH/MBL2** | **ribosome** | **RPS3/RPL35A/RPL18A/RPS2/RPL13/GADD45GIP1/MRPS34/MRPL11/NCK1/RPL37/RPL28/RPSA/RPL7A/GCN1/RPL3L/AURKAIP1/ZCCHC17/RPL41/MRPL38/MRPL23/RPL19/MRPL55/RPS17/MRPS2/MRPL21/MPV17L2/MRPS15/MRPL14/EIF2AK4/EIF2A/RPS4X/RPS16/RPL11/RPS28/MRPL34/UBA52/NDUFA7/FXR2/MRPL54/MRPL24/MRPS18C/RPL24/MRPL41/MRPS6/RPL35/MRPL45/RPL17/RPS23/NSUN3/MTERF4/MRPS35/APOD/MRPL18/RPL23/RPS18/RPL34/DAP3/RPL22/RPL26/NUFIP2/MRPS21/RPL5/RPS20/MRPS12/RPL36/RPS21/MT3/RPL10L/RPL39/MRPL46/RPL13A/SERP1/MRPL30/LARP1/MRPS9/RPL4/RPS27/RPS11/MRPL1/MTG1/DNAJC21/RPL30/RPL10A/RSL24D1/RPS4Y1/MRPL53/MRPL4/MRPL13/BTF3/MRPL39/EIF2AK2/MTG2/RPL23A** |
|  |  | **phagocytic cup** | **TNF/TLR4** | **arge ribosomal subunit** | **RPL35A/RPL18A/RPL13/GADD45GIP1/MRPL11/RPL37/RPL28/RPL7A/RPL3L/ZCCHC17/RPL41/MRPL38/MRPL23/RPL19/MRPL55/MRPL21/MPV17L2/MRPL14/RPL11/MRPL34/UBA52/FXR2/MRPL54/MRPL24/RPL24/MRPL41/RPL35/MRPL45/RPL17/NSUN3/MTERF4/MRPL18/RPL23/RPL34/RPL22/RPL26/RPL5/RPL36/RPL10L/RPL39/MRPL46/RPL13A/MRPL30/RPL4/MRPL1/RPL30/RPL10A/MRPL53/MRPL4/MRPL13/MRPL39/RPL23A** |
|  |  | **plasma membrane signaling receptor complex** | **CSF2/TRBV11-2/TLR2/IL6R** | **coated vesicle** | **AP2M1/CTSZ/SLC18A1/VPS41/SH3GL2/EPN1/SLC32A1/HBEGF/RAB27A/ATP6V1F/VPS16/HLA-E/HLA-DPA1/ATP6V1H/SCAP/HLA-DRA/STX5/KLHL12/VTI1A/STEAP2/ROR2/RGS19/AP1S1/HIP1/TMED4/SEC22B/IDUA/VAMP8/LMAN2/ERGIC3/AP2S1/DAB2/ARCN1/DENND1A/SLC18A3/HLA-DPB1/RNASEK/CNIH2/RAB5A/EPN3/KDELR2/B2M/VWF/COPE/ASTN2/ATP6V0E2/ATP6V0B/PANK1/HEATR5B/BTC/SFTA3/SLC17A7/SCYL1/RAB3A/CLTB/ATP6V1B2/YIPF5/FCHO1/SNX9/CD3D/SEC16A/PEF1/ADAM10/TFRC/GAD2/SYNRG/TMED3/YIF1A/AP3B2/SEC13/CCDC115/VPS11/HLA-DQB2/BCAP31/LDLR/AP1B1/DENND1C/VPS18/ATP6V1C1/PDCD6/HLA-A/CD59/CD207/MYO6/AP1S2/VPS33B/PHETA2/PACSIN1/DDHD2/AP4B1/HLA-DRB1/HLA-DQB1/HIP1R/CNIH4/KDELR3/TEPSIN/ATP6V1D/SLC2A8/AP2B1/DNAJC5/RAB27B/NECAP2/FZD5/GOPC/HLA-DQA2/LMAN1/REEP6/CHRM2/CD74/TMED5/SFTPD/SCARB2/CLRN1/SEC23A/OCRL/NUMB/APOB/AP1M2/AP1G1/YIF1B** |
|  | **MF** | **NAD+ nucleosidase activity** | **TLR4/IL1RL2/TLR2/IL1R1** | **iron-sulfur cluster binding** | **ISCU/RPS3/AIFM3/NDUFV1/FECH/MOCS1/POLD1/NDUFS2/ABAT/ISCA1/RFESD/BRIP1/EXO5/KIF4A/TYW1B/GLRX2/ACO1/SDHB/NUBP1/CISD1/NDUFV2/TYW1/KIF4B/MUTYH/ACO2/CDK5RAP1/PPAT/DPH2/PRIM2/XDH/DDX11/NDUFS8/GLRX3/FDX1/ELP3** |
|  |  | **NAD(P)+ nucleosidase activity** | **TLR4/IL1RL2/TLR2/IL1R1** | **metal cluster binding** | **ISCU/RPS3/AIFM3/NDUFV1/FECH/MOCS1/POLD1/NDUFS2/ABAT/ISCA1/RFESD/BRIP1/EXO5/KIF4A/TYW1B/GLRX2/ACO1/SDHB/NUBP1/CISD1/NDUFV2/TYW1/KIF4B/MUTYH/ACO2/CDK5RAP1/PPAT/DPH2/PRIM2/XDH/DDX11/NDUFS8/GLRX3/FDX1/ELP3** |
|  |  | **NAD+ nucleotidase, cyclic ADP-ribose generating** | **TLR4/IL1RL2/TLR2/IL1R1** | **K48-linked polyubiquitin modification-dependent protein binding** | **UBXN1/MINDY2/NPLOC4/MINDY1/IKBKE/UBQLN4/UFD1/RNF31** |
|  |  | **cytokine activity** | **TNF/CSF2/IL1B/IFNG/HMGB1/IFNB1/IL11** | **cadherin binding** | **PPP1CA/PAK6/EIF4H/SND1/CC2D1A/CTNNA2/PRDX1/CEMIP2/RPS2/TRIM25/VCL/TMPO/PTPRH/HNRNPK/NCK1/CAST/EEF1D/RPL7A/GCN1/STX5/CRKL/RSL1D1/TES/ATIC/EPS15L1/AHNAK/ANLN/SLC3A2/MMP24/EXOC3/TWF2/ARFIP2/PFKP/EIF2A/LRRC59/EEF2/MARK2/FER/PTPRO/ASAP1/GLOD4/NUDC/EMD/SH3GLB2/ALDOA/PARVA/ANXA2/TJP1/GAPVD1/PSEN1/FXYD5/RPL24/PI4KA/TRIM29/YWHAB/ABCF3/CORO1B/SCYL1/BAIAP2/TBC1D10A/CHMP4B/SNX9/RUVBL1/PLCB3/FASN/RPL34/PRDX6/ANK3/TLN1/TNKS1BP1/CSNK1D/LDHA/CAPZB/EPS8L1/DIAPH3/PARK7/DNAJB1/EIF4G2/KLC2/CAPG/PACSIN2/CDH22/ARHGAP1/PTPRJ/LARP1/ANXA1/JUP/PKP2/MYO1B/NOP56/EFHD2/OLFM4/PPME1/PDLIM5/RANGAP1/DCHS1/GIPC1/PLEC/CDH11/ARHGAP18/MICALL1/TMOD3/ZC3H15/RAB11B/CKAP5/ARVCF/WASF2/CDH1/EPCAM/AHSA1/NUMB/SHTN1/ESYT2/UBFD1/CDH13/USP8/YWHAZ/DOCK9/RPL23A/CBL/SNX2/VAPB/CDC42EP1** |
|  |  | **cytokine receptor binding** | **TNF/CSF2/IL1B/IFNG/IFNB1/IL11/IL6R** | **ribosomal small subunit binding** | **ERAL1/MTIF3/EIF1/UNG/NME1/CPEB2/LARP1/EIF1B/PIM1/NPM1** |
| **KEGG** | | **Coronavirus disease - COVID-19** | **TNF/CSF2/IL1B/TMPRSS2/TLR4/MBL2/TLR2/IFNB1/IL6R** | **Pathways of neurodegeneration - multiple diseases** | **NDUFA4L2/DVL3/HTRA2/MAP2K3/CYBB/NOS2/DCTN3/APC2/NDUFB11/CSNK1A1L/NDUFV1/ATP5MC1/TRPC3/ATF4/GRM5/BAK1/GSK3B/UBE2G2/VDAC2/C9orf72/TPTEP2-CSNK1E/DNAI1/RAC1/KLC1/NDUFB7/TUBA1A/NEFM/RELA/CACNA1S/TUBA3C/NDUFS2/PIK3C3/CSNK2B/CACNA1B/NDUFAB1/HIP1/ATP5PO/PSEN2/WNT7A/FZD10/PIK3R4/NDUFA11/NDUFS3/NDUFA3/DCTN2/TOMM40/COX8A/CAPN1/SLC25A6/UBA52/STX1A/TNFRSF1B/SLC25A4/DLG4/RAB5A/NDUFA7/PSMD4/COX5B/CAT/PLCB2/WNT10B/BAD/PSMC3/DCTN6/PSMA4/PSEN1/NDUFS5/DNAH17/NDUFB9/UQCRC2/UBE2L3/NDUFA2/SOD1/ARAF/CYC1/SDHB/HSD17B10/PPP3CC/WNT3/MAP3K10/NDUFB10/NEFL/SLC25A5/NDUFS4/PSMA2/LRP5/PDYN/UQCRQ/ATP2A1/TUBB3/PSMD11/PLCB3/CHRNA7/MAP2K1/MAPK12/CAMK2D/WNT16/NDUFV3/PSMB7/FZD3/WNT6/NDUFB8/CAMK2G/DAXX/SLC25A31/UBA7/MAP2K7/PARK7/NDUFV2/SNCA/TRAP1/PPP3R1/TUBA4A/PLCG1/UBC/TUBB/CYCS/RAB39B/VDAC3/KLC2/ATG101/PSMB1/ATP5F1D/NDUFA4/TRAF2/GPX3/GRM1/UCHL1/PPID/SEM1/MAPK10/PSMC1/UBE2L6/FIG4/PLCB1/PPIF/ATP5F1C/BCL2L1/TANK/NDUFA10/DNALI1/PSMC2/NDUFB5/PSMD7/TUBB4B/SDHA/COX7C/ACTR10/DKK1/DNAL1/PSMA7/COX6B1/RYR3/FZD5/NDUFS8/WNT1/COX6A1/MAP2K2/GRIN2B/FADD/IL1A/COX7B/TUBB1/BCL2/GRIA4/CASP7/NDUFC2-KCTD14/MAP2K6/FRAT2/CALM1/SDHD/COX4I1/PSMA1/GRIN2D/ATG2B/VAPB** |
|  |  | **Cytokine-cytokine receptor interaction** | **TNF/CSF2/IL1B/IL1RL2/IFNG/IFNB1/IL1R1/IL11/IL6R** | **Alzheimer disease** | **NDUFA4L2/DVL3/CYBB/NOS2/APC2/NDUFB11/CSNK1A1L/NDUFV1/ATP5MC1/ATF4/GRM5/GSK3B/VDAC2/TPTEP2-CSNK1E/KLC1/NDUFB7/TUBA1A/RELA/CACNA1S/TUBA3C/NDUFS2/PIK3C3/CSNK2B/SLC39A8/NDUFAB1/ATP5PO/PSEN2/WNT7A/FZD10/PIK3R4/NDUFA11/NDUFS3/NDUFA3/PIK3CB/COX8A/CAPN1/SLC25A6/SLC25A4/NDUFA7/PSMD4/COX5B/PLCB2/SLC39A4/WNT10B/BAD/PSMC3/PSMA4/PSEN1/NDUFS5/APH1A/NDUFB9/UQCRC2/NDUFA2/ARAF/CYC1/SDHB/HSD17B10/PPP3CC/WNT3/NDUFB10/SLC25A5/NDUFS4/PIK3R3/PSMA2/LRP5/PIK3R1/SLC11A2/ADAM10/AKT3/UQCRQ/ATP2A1/TUBB3/PSMD11/PLCB3/CHRNA7/MAP2K1/IRS2/WNT16/SLC39A12/NDUFV3/PSMB7/SLC39A1/FZD3/WNT6/NDUFB8/SLC25A31/MAP2K7/NDUFV2/SNCA/PPP3R1/TUBA4A/AKT2/PSENEN/TUBB/CYCS/VDAC3/KLC2/ATG101/PSMB1/ATP5F1D/NDUFA4/TRAF2/PPID/SEM1/MAPK10/PSMC1/PLCB1/PPIF/ATP5F1C/NDUFA10/PSMC2/NDUFB5/PSMD7/TUBB4B/SDHA/COX7C/DKK1/PSMA7/COX6B1/MME/RYR3/FZD5/NDUFS8/WNT1/COX6A1/MAP2K2/GRIN2B/FADD/APH1B/IL1A/COX7B/TUBB1/CASP7/LPL/NDUFC2-KCTD14/EIF2AK2/FRAT2/CALM1/SDHD/COX4I1/GAPDH/PSMA1/GRIN2D/ATG2B** |
|  |  | **Rheumatoid arthritis** | **TNF/CSF2/IL1B/TLR4/IFNG/CD28/TLR2/IL11** | **Parkinson disease** | **NDUFA4L2/HTRA2/GNAS/SLC18A1/ADORA2A/NDUFB11/NDUFV1/ATP5MC1/ATF4/UBE2G2/VDAC2/KLC1/NDUFB7/TUBA1A/TUBA3C/NDUFS2/SLC39A8/NDUFAB1/ATP5PO/NDUFA11/NDUFS3/NDUFA3/COX8A/SLC25A6/UBA52/SLC25A4/NDUFA7/PSMD4/COX5B/SLC39A4/PSMC3/PSMA4/NDUFS5/NDUFB9/UQCRC2/UBE2L3/NDUFA2/SOD1/DRD2/CYC1/SDHB/TXN2/NDUFB10/SLC25A5/NDUFS4/PSMA2/SLC11A2/UQCRQ/TUBB3/PSMD11/CAMK2D/SLC39A12/NFE2L2/NDUFV3/PSMB7/SLC39A1/NDUFB8/CAMK2G/DAXX/SLC25A31/UBA7/PARK7/NDUFV2/SNCA/TRAP1/TUBA4A/PLCG1/UBC/TUBB/CYCS/VDAC3/KLC2/PSMB1/ATP5F1D/NDUFA4/UCHL1/SEM1/MAPK10/PSMC1/UBE2L6/PPIF/ATP5F1C/BCL2L1/NDUFA10/PSMC2/NDUFB5/PSMD7/DRD1/TUBB4B/SDHA/COX7C/PSMA7/COX6B1/RYR3/NDUFS8/COX6A1/PRKACB/GNAI3/COX7B/TUBB1/DUSP1/NDUFC2-KCTD14/CALM1/MAOA/SDHD/COX4I1/PSMA1** |
|  |  | **Chagas disease** | **TNF/IL1B/TLR4/IFNG/TLR2/IFNB1/SERPINE1** | **Prion disease** | **NDUFA4L2/CYBB/NDUFB11/NDUFV1/ATP5MC1/ATF4/GSK3B/VDAC2/RAC1/KLC1/NDUFB7/TUBA1A/CACNA1S/TUBA3C/NDUFS2/CSNK2B/CACNA1B/NDUFAB1/ATP5PO/NDUFA11/NDUFS3/C7/NDUFA3/PIK3CB/COX8A/SLC25A6/SLC25A4/NDUFA7/NOTCH1/PSMD4/COX5B/C8A/BAD/PSMC3/PSMA4/NDUFS5/NDUFB9/UQCRC2/NDUFA2/SOD1/CYC1/SDHB/PPP3CC/CAV2/NDUFB10/SLC25A5/NDUFS4/PIK3R3/PSMA2/PIK3R1/EGR1/UQCRQ/TUBB3/PSMD11/MAPK12/NDUFV3/PSMB7/NDUFB8/SLC25A31/NDUFV2/PPP3R1/TUBA4A/TUBB/CYCS/CCL5/VDAC3/KLC2/PSMB1/ATP5F1D/NDUFA4/CREB5/CREB3L3/SEM1/MAPK10/PSMC1/NCAM1/PPIF/ATP5F1C/CYBA/NDUFA10/C1QB/PSMC2/NDUFB5/PSMD7/CREB3L2/TUBB4B/SDHA/COX7C/PSMA7/COX6B1/HSPA1B/RYR3/NDUFS8/COX6A1/GRIN2B/PRKACB/IL1A/COX7B/TUBB1/NDUFC2-KCTD14/HSPA1L/NCF4/SDHD/COX4I1/PSMA1/GRIN2D** |
|  |  | **Amoebiasis** | **TNF/CSF2/IL1B/TLR4/IFNG/TLR2/IL1R1** | **Diabetic cardiomyopathy** | **NDUFA4L2/PPP1CA/CD36/CYBB/NDUFB11/NDUFV1/TBC1D4/ATP5MC1/GSK3B/VDAC2/RAC1/NDUFB7/RELA/NDUFS2/NDUFAB1/NOS3/ATP5PO/SLC2A1/GSR/NDUFA11/NDUFS3/NDUFA3/PIK3CB/COX8A/SLC25A6/SLC25A4/COL3A1/NDUFA7/COX5B/PLCB2/NDUFS5/NDUFB9/UQCRC2/MPC2/MPC1/NDUFA2/MMP9/CYC1/SDHB/NDUFB10/SLC25A5/NDUFS4/PIK3R3/PIK3R1/AKT3/PPP1CC/PARP1/UQCRQ/ATP2A1/PLCB3/MAPK12/CAMK2D/NDUFV3/NDUFB8/CAMK2G/SLC25A31/NDUFV2/AKT2/VDAC3/ATP5F1D/NDUFA4/PDK2/MAPK10/PDK3/PLCB1/PPIF/ATP5F1C/CYBA/NDUFA10/NDUFB5/MPC1L/SDHA/COX7C/COX6B1/NDUFS8/COX6A1/PTPA/COX7B/NDUFC2-KCTD14/NCF4/SDHD/COX4I1/GAPDH** |

BP: Biological Process

MF: Molecular Function

CC: Cellular Component
